# Advanced Platelet-Rich Fibrin and Connective Tissue Graft for Treating Marginal Tissue Recessions: A Randomized, Controlled Split-Mouth Study

**DOI:** 10.7759/cureus.35761

**Published:** 2023-03-04

**Authors:** Mahmoud Abu-Ta'a

**Affiliations:** 1 Dentistry, Arab American University, Ramallah, PSE

**Keywords:** root coverage, gingival recession, advanced platelet-rich fibrin, coronally advanced flap, connective tissue graft

## Abstract

Objectives: This study aimed to evaluate and compare the clinical outcomes of advanced platelet-rich fibrin (A-PRF) and connective tissue graft (CTG) in treating marginal tissue recessions.

Materials & Methods: Fifteen patients with isolated bilateral maxillary gingival recessions were recruited for the study, with 30 defects. The defects were classified as Miller's class I/II gingival recession on the canine or premolar region. Patients were randomly divided into two groups, each receiving one of the two treatment techniques (A-PRF or CTG) on a different side of the maxilla in a split-mouth design. Clinical parameters such as recession height (RH), recession width (RW), probing pocket depth (PPD), clinical attachment level (CAL), a width of attached gingiva (WAG), and keratinized tissue height (KTH) were evaluated at baseline, 3, and 6 months. Changes in biotype, Recession Esthetic Score (RES), and Visual Analogue Score-Esthetics (VAS-E) were also evaluated at 6 months.

Trial Registration: Ethics approval number (Helsinki): PHRC/HC/877/21 and registered at the Clinical Trials Registry under the number NCT05267015

Results: At the end of 6 months, there was a statistically significant reduction in RH and RW in both groups, with the mean RC% of 69.2±22.91, and 88.66±33.18 in Groups I and II, respectively. Intergroup analysis showed statistically significant differences in recession parameters between groups at 3 and 6 months, with better outcomes for the CTG group.

Conclusions: This study demonstrates that A-PRF and CTG effectively manage gingival recession defects. However, CTG resulted in better clinical outcomes in terms of reduction in recession height and width.

## Introduction

Gingival recession is a common periodontal condition characterized by root surface exposure due to soft and hard tissue loss. It can lead to functional and aesthetic problems, such as increased tooth sensitivity, root caries, and an altered smile appearance [1]. Gingival recession is on the rise globally, and studies have reported that 40-68% of individuals have one or more gingival recession defects [2,3].

Over the years, many surgical treatments have been proposed to treat gingival recessions, including pedicle flaps [4], free gingival grafts [5], coronally advanced flaps [6], tunneling techniques [7], subepithelial connective tissue grafts [8], guided tissue regeneration [9], enamel matrix derivative [10], hyaluronic acid [11], and acellular dermal matrix [12]. The most commonly used and researched technique combines coronally advanced flaps and connective tissue grafts [13]. However, this technique can be technically demanding and requires a second surgical site, increasing the risk of morbidity. Platelet-rich fibrin (PRF) has recently been proposed as an alternative treatment option for gingival recession. It does not require a second surgical site and is minimally invasive. PRF is a biological matrix rich in growth factors, platelets, and leukocytes that can accelerate and improve soft-tissue healing [14].

The use of PRF has been extensively studied in the oral and maxillofacial surgery, orthopedics, and plastic surgery fields, and the results have been encouraging. The advanced PRF (A-PRF) is a modification of the classical PRF protocol, characterized by a higher release of growth factors, especially during the first ten days [15]. The A-PRF has been shown to have a more potent effect on the healing process than the classical PRF. Additionally, A-PRF is easy to prepare and can be used in many different surgical procedures [16,17].

This randomized, controlled split-mouth study aimed to evaluate and compare the clinical effectiveness of the CAF+A-PRF and CAF+CTG techniques in the treatment of isolated bilateral maxillary gingival recessions. The study used the latest technology and research methods to provide accurate and up-to-date results. By comparing the two treatment options, the study could help dental professionals to decide which treatment option is more suitable for their patient's needs and goals. The study would also contribute to understanding the role of growth factors in the healing process and their potential use in regenerative dentistry.

This study aimed to test the null hypothesis that there would be no difference in the mean change in the reduction of recession height (RH), recession width (RW), probing pocket depth (PPD), clinical attachment level (CAL), a width of attached gingiva (WAG), keratinized tissue height (KTH), changes in biotype, recession esthetic score (RES), and Visual Analogue Score-Esthetics (VAS-E) between Group I (CAF+A-PRF) and Group II (CAF+CTG) after 3 and 6 months.

This article was previously posted to the Research Square preprint server on June 4, 2022.

## Materials and methods

This study adhered to the Consolidated Standards of Reporting Trials (CONSORT) statement for reporting randomized controlled trials. This randomized, controlled split-mouth clinical study was conducted at the Department of Dental Sciences, Faculty of Graduate Studies, Arab American University of Palestine. The study protocol was approved by the ethical committee of the Palestinian Health Research Council (Approval number PHRC/HC/877/21), was performed following the Declaration of Helsinki in 1975 and revised in Tokyo in 2004, and registered at the Clinical Trials Registry under the number NCT05267015. The study purpose and clinical procedures were explained to the patients, who gave their written informed consent before initiating the procedure.

Selection of Participants: Fifteen patients with isolated bilateral gingival recession (n=30 recession defects) were selected according to inclusion and exclusion criteria.

Inclusion Criteria: Patients with bilateral isolated gingival recession, Miller's class I/II affecting canines or premolars, recession sites associated with probing depth <4mm, sound teeth, healthy gingiva, and immobile teeth.

Exclusion Criteria: Medically compromised patients, pregnant females, heavy smokers (>10 cigarettes), recession sites associated with probing depth ≥4 mm, abrasion/caries and mobility, and patients with improper oral hygiene.

Sample size determination

For the present study to ascertain the sample size (number of sites to be treated in each group), pre-hoc analysis with the following method was done:

n = 2× (Zα/2 + Zβ)2 SD2 /d2 (where n: sample size per group; SD: pooled standard deviation being 0.5 in this; d: difference in the means (effect size); Zα/2: significance level, Zβ: the power of the study). Assuming 80% power, a 5% significance level with a 95% confidence interval, and a standard of 0.50, the required sample size per group is 13 subjects in each group. Assuming a 20% loss to follow-up, the final sample size is 15 in each group.

A total of 15 patients presenting with 30 gingival recession defects in maxillary canines or premolars fulfilling the study criteria were enrolled. The following parameters were evaluated during the study period (baseline, 3 months, and 6 months): Recession Height (RH), Recession Width (RW), Width of attached Gingiva (WAG), and Keratinized Tissue Height (KTH), Probing Pocket Depth (PPD), Clinical Attachment Level (CAL) and gingival biotype. The percentage of root coverage (%RC) was calculated at the end of 3 & 6 months. Recession Esthetic Score (RES) and Visual Analogue scale to evaluate Esthetics (VAS-E) were evaluated at the end of 6 months.

Sample distribution

**Figure 1:**
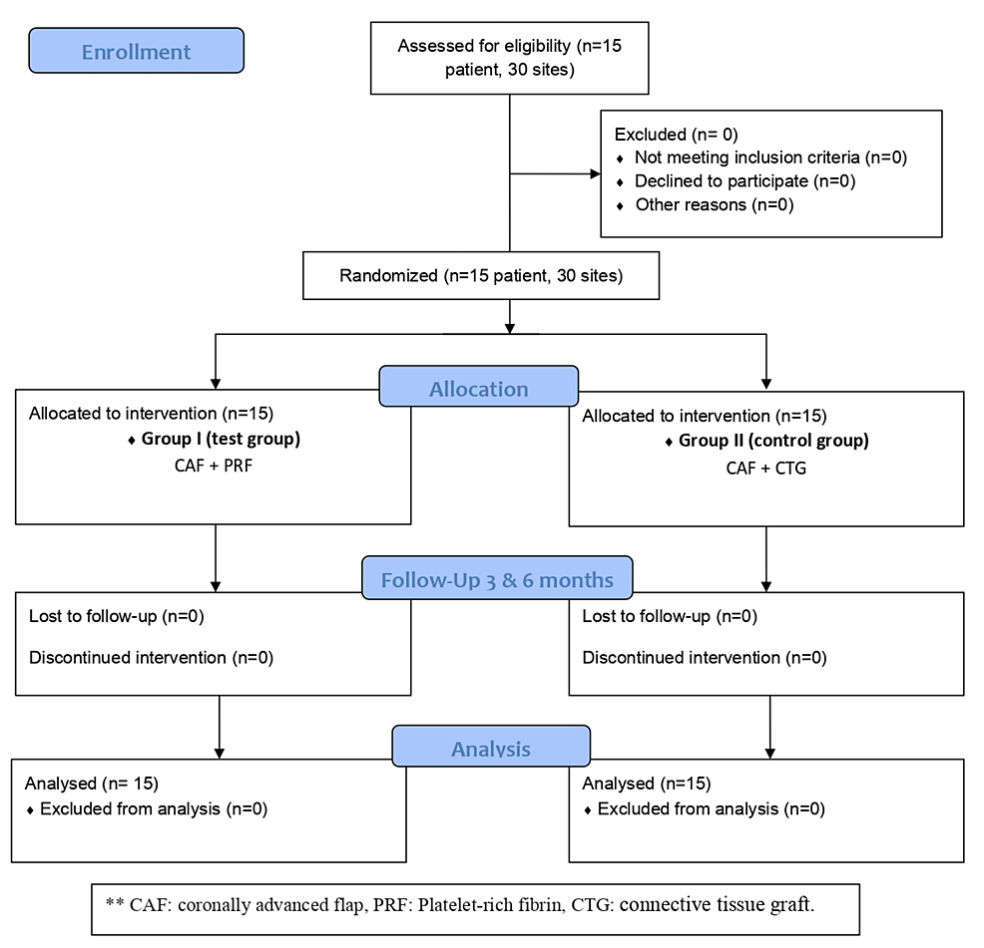
Consort Flowchart of the study CAF: coronally advanced flap, PRF: Advanced Platelet-Rich Fibrin, CTG: connective tissue graft

This clinical study was designed as a split-mouth, randomized, controlled clinical trial. Bilateral gingival recession defects were randomly assigned to the test (CAF + A-PRF) or the control (CAF+ CTG) groups using a computer-generated randomization table; these groups were left and right sides of the patient's mouth (split-mouth design) in which each patient was treated with two techniques as follows; test group or Group I (n=15 defects; 7 left sides & 8 right sides) were treated by coronally advanced flap with advanced platelet-rich fibrin (A-PRF). In contrast, the control group or Group II (n=15; 8 right sides & 7 left sides) was treated by coronally advanced flap with connective tissue graft (CTG) (Figure 1).

Surgical procedures

All patients received oral hygiene instructions and were enrolled when plaque indices were below 20% [18]. Occlusal adjustments were made to eliminate premature contacts and interferences. All surgeries were performed by the same expert periodontist (MAT). The recession defect sites were prepared according to the surgical technique described by Langer and Langer [8].

After applying local anesthesia using 2% lidocaine (epinephrine at 1:100,000), sulcular incisions were made on the recipient's teeth and joined to horizontal incisions extending into the adjacent interdental areas slightly coronal the CEJ. Two vertical incisions that began at the adjacent teeth' line angles were connected to the horizontal incisions resulting in a trapezoidal-shaped flap. This partial-thickness flap design provided a vascular connective tissue bed for the selected graft material. All recession defects were scaled entirely using. Gracey curettes (Gracey Curettes, Hu-Friedy, Chicago, IL, USA). At all recession sites, both grafts extended apically beyond the apical base of the recession defect by > 2mm.

At the test site (CAF+ A-PRF), an A-PRF membrane of 2 mm thickness was prepared using Choukroun's A-PRF™ protocol [19] (1300 rpm for 14 minutes, using red cap glass tubes) and at the control site (CAF + CTG) a connective tissue graft of 1.5 mm thickness was harvested from the palate. Both grafts were placed over the defects, according to the randomization table. They were stabilized to the interdental papillae and adjacent soft tissue at the apical part with horizontal mattress sutures using Monocryl® 5-0 absorbable sutures (Ethicon™, Cornelia, Georgia, USA). Then, each partial-thickness flap was released, repositioned over the graft and beyond the CEJ, and stabilized using Ethilon® Nylon 5-0 nonabsorbable suture (Ethicon™, Cornelia, Georgia, USA). Gentle pressure was applied for 5 minutes to minimize the thickness of the clot.

Post-operative management

Patients were given antibiotics and anti-inflammatory medication for five days postoperatively. Patients were instructed to refrain from brushing the surgical sites for 2 weeks. They were instructed to use 0.2% Chlorhexidine digluconate-containing mouth rinses (Gargarol, Beit Jala Pharmaceutica Co., Beit Jala, Palestine) during the first 2 weeks postoperatively. Sutures were removed after 2 weeks, and the patients started mechanical plaque control using a soft surgical brush for two weeks. Healing was uneventful, without any post-operative complications such as graft infection, rejection, or wound dehiscence. Regular tooth brushing was resumed at 28 days postoperatively.

The patients were given a follow-up schedule, and recall visits were scheduled for 1, 3, and 6 months postoperatively. At each visit, the patients were evaluated for the following parameters; recession height, recession width, probing pocket depth, clinical attachment level, a width of attached gingiva, keratinized tissue height, gingival biotype, recession esthetic score, and visual analog scale to evaluate esthetics.

Statistical analysis

All data were analyzed using IBM® SPSS® version 22.0 (SPSS Inc., Chicago, IL, USA). Descriptive statistics were used to describe the baseline characteristics of the study groups and were expressed as mean ± standard deviation. The primary outcome variable in this study was the reduction in recession height (RH). The secondary outcome variables were the reduction in recession width (RW), probing pocket depth (PPD), clinical attachment level (CAL), a width of attached gingiva (WAG), keratinized tissue height (KTH), changes in biotype, Recession Esthetic Score (RES), and Visual Analogue Score-Esthetics (VAS-E). The normality of data distribution was tested using the Shapiro-Wilk test. After the normality assessment, the data were analyzed with the Mann-Whitney U, and Kruskal-Wallis ANOVA tests to assess intergroup differences. The intragroup differences were analyzed using paired sample t-tests for normally distributed continuous variables and the Wilcoxon signed-rank test for non-normally distributed continuous variables. The level of significance was set at p<0.05.

## Results

A total of 15 patients (12 males and 3 females) representing 30 isolated gingival recession defects distributed between the right and left quadrants of the maxilla completed the study (Figure 1). All 30 recession defects were available for analysis comprising 73.3% Miller's class I and 26.7% class II in the test group and 66.7% Miller's class I and 33.3% class II in the control group. Baseline data were homogenous for both groups (Table 1). At baseline, the two study groups had no statistically significant differences in RH, RW, PPD, CAL, WAG, KTH, and gingival biotype values.

**Table 1:** The mean descriptive parameters among the study groups at baseline Significant at *p<0.05*, RH: Recession width, RW: Recession width, PPD: Pocket probing depth, CAL: Clinical attachment loss, WAG: Width of attached gingiva, KTH: Keratinized tissue height.

Parameter	CAF+A-PRF	CAF+CTG	p-value
AGE (years)	39.47 ± 6.69	34.68 ± 8.6	0.076
GENDER	80% Male	20% Female	1
Quadrant	53.33% Q1	46.67% Q1	0.553
	46.67% Q2	53.33% Q2	0.553
MILLER	73.3% Class I	66.7% Class I	0.507
	26.7% Class II	33.3% Class II	0.507
RH (mm)	2.53 ± 0.74	2.87 ± 0.83	0.308
RW (mm)	3.40 ± 0.63	3.87 ± 0.51	0.303
PPD (mm)	1.10 ± 0.63	1.07 ± 0.25	0.292
CAL (mm)	4.13 ± 1.12	3.93 ± 0.96	0.263
WAG (mm)	2.60 ± 0.73	2.40 ± 0.50	0.322
KTH (mm)	3.20 ± 0.67	3.47 ± 0.64	0.567
BIOTYPE	73.3% Thick	66.7% Thick	0.507
	26.7% Thin	33.3% Thin	0.507

Both interventions resulted in a statistically significant improvement in all clinical parameters (p-value <0.05) from baseline to 3 and 6 months. However, Group II (CAF+CTG) improved all clinical parameters better than group I (CAF+A-PRF).

Intragroup analysis showed that, by the end of 6 months, the mean gingival recession height and width significantly reduced in both groups, with a mean root coverage percentage of 69.2±22.91 and 88.66±33.18 in groups I and II, respectively (Table 3). Pair-wise analysis showed a statistically significant difference (p<0.05) between group I and group II at 3 and 6 months in all parameters, except PPD and KTH at 3 months (Table 2, 3).

**Table 2:** Clinical parameters at 3 and 6 months Significant at *P* <0.05, RH: Recession width, RW: recession width, RC%: root coverage percentage, PPD: pocket probing depth, CAL: clinical attachment loss, WAG: width of attached gingiva, KTH: keratinized tissue height, RES: recession esthetic score, VAS: visual analogue scale.

Parameters	CAF+A-PRF	CAF+CTG	3 Months p-value	6 Months p-value
RH (mm)	0.87 ± 0.83	0.27 ± 0.70	0.031	0.031
RW (mm)	1.40 ± 1.24	0.53 ± 1.4	0.045	0.045
RC%	69.2 ± 22.91	88.66 ± 33.18	0.035	0.037
PPD (mm)	1.20 ± 0.25	1.17 ± 0.25	0.091	0.04
CAL (mm)	1.33 ± 0.96	1.93 ± 0.72	0.019	0.025
WAG (mm)	2.53 ± 0.64	3.20 ± 0.77	0.011	0.007
KTH (mm)	3.67 ± 0.48	4.27 ± 0.79	0.06	0.017T
Thick Biotype	20%	66.7%	NA	0.047
Res Aesthetic Scores	8.27 ± 1.43	9.40 ± 1.24	0.052	NA
VAS Aesthetic Scores (E)	8.67 ± 0.90	9.00 ± 1.41	0.008	NA

**Table 3:** Comparison of Baseline and Follow-Up clinical measurements (3 and 6 Months) . Significant at P <0.050, RH: Recession width, RW: Recession width, RC%: Root coverage percentage, PPD: Pocket probing depth, CAL: Clinical attachment loss, WAG: Width of attached gingiva, KTH: Keratinized tissue height, RES: Recession esthetic score, VAS: Visual Analogue scale

Parameter	Z-Value (Baseline vs 3 Months)	p-Value (Baseline vs 3 Months)	Z-Value (Baseline vs 6 Months)	p-Value (Baseline vs 6 Months)
RH	-0.979	0.308	-2.299	0.031
RW	-2.281	0.303	-2.101	0.045
PPD	-2.751	0.292	0.000	0.040
CAL	-0.607	0.263	-2.030	0.025
WAG	-2.950	0.322	-2.296	0.011
KTH	-0.891	0.567	-2.821	0.017
RC%	NA	NA	-2.140	0.037
RES AESTHETIC SCORES	NA	NA	-2.341	0.041
VAS AESTHETIC SCORES (E)	NA	NA	-1.367	0.008

At 3 months, a significant difference was found between the two groups for RH (p=0.031), RW (p=0.045), RC% (p=0.035), CAL (p=0.019), and WAG (p=0.011). The mean values for RH, RW, and RC% were higher in Group II compared to Group I. The mean values for CAL and WAG were higher in group I compared to Group II. No significant difference was found between the two groups for KTH (p=0.060) and PPD (p=0.091). At 6 months, a significant difference was found between the two groups for RH (p=0.031), RW (p=0.045), RC% (p=0.037), PPD (p=0.040), CAL (p=0.025), WAG (p=0.007), and KTH (p=0.017). The mean values for RH, RW, RC%, and PPD were higher in Group II compared to Group I. The mean values for CAL and WAG were higher in group I compared to Group II. The mean values for KTH were higher in Group II compared to Group I.

At 6 months, the healed recession defects displayed a significantly higher percentage of thick gingival biotype in the control group compared to the test group (p=0.047). At the end of 6 months, the results showed that the CAF+CTG group (Group II) had a higher mean RES score (9.40 ± 1.24) compared to the CAF+A-PRF group (Group I) (8.27 ± 1.43). The results also showed that the CAF+CTG group had a higher mean VAS-E score (9.00 ± 1.41) than the CAF+A-PRF group (8.67 ± 0.90). Pair-wise analysis showed a statistically significant difference between the two groups regarding VAS-E scores.

The probing pocket depth (PPD) results indicate no statistically significant difference between the two groups (p-value = 0.292) at the baseline measurement. However, after 3 months, the test group showed a statistically significant increase in PPD compared to the control group (p-value = 0.040). At 6 months, the two groups had no significant difference in PPD (p-value = 0.091). Regarding the post-operative period, all recipient sites in both test and control groups were healed uneventfully. No severe post-operative complications were reported, such as bleeding or graft exposure.

## Discussion

Root coverage of gingival recession defects is still challenging in periodontal treatment due to its high prevalence, surgical morbidity, and the expertise needed to harvest a CTG sufficient to yield a predictable treatment [20]. Thus, evaluating the efficacy of substitutes for CTG in periodontal plastic surgery is essential.

To the best of our knowledge, the present study represents the first clinical trial using a split-mouth design to test whether CAF + A-PRF could be a successful treatment option when compared to CAF + CTG in the treatment of maxillary localized gingival recession defects. In the present study, the CAF + A-PRF demonstrated significant improvement in all clinical parameters compared to the baseline. In particular, RH and RW were significantly reduced over the 3 and 6-month follow-ups. However, CAF + CTG showed a better RC% and better improvement in all clinical parameters over CAF + A-PRF.

The present clinical trial followed a split-mouth design to test the efficiency of CAF in combination with A-PRF compared to CAF in combination with CTG, which means all patients received both interventions. The split-mouth design is the best study type to investigate two surgical treatments in the oral cavity. This study type reduces interindividual variability between groups, potentially affecting the baseline status and the response to therapy. As well this study type can improve the data analysis. Moreover, the CAF was standardized as the flap design and CTG as the positive control as they are the gold standard and the most predictable treatment option for root coverage [21]. This surgical protocol's choice led to excellent clinical outcomes observed at 6 months, with almost 90% of root coverage for CAF + CTG and about 70% for CAF + A-PRF. However, the %RC after 6 months was still within the interval of reported values (64% to 96%), demonstrating the effectiveness of our surgical protocol [22]. 

In the present study, the mean baseline RW value in the test group was lower than in the control group. However, at 6 months, the mean RW value in the test group was higher than that in the control group though the control group showed more RC%. This is different from previously published literature, where it has been mentioned that the wider the RW, the less root coverage should be expected [23]. Nevertheless, CAF + A-PRF might represent an alternative treatment option to the CAF + CTG surgical technique in treating maxillary localized gingival recession defects. 

Considering the null hypothesis of this study, the results showed that both groups had a statistically significant reduction in RH, RW, and RC% at 3 months (p-value=0.031, 0.045, and 0.035 respectively) and 6 months (p-value=0.031, 0.045, and 0.037 respectively). However, the mean reduction in RH and RW was more significant in Group II (CAF+CTG) compared to Group I (CAF+A-PRF). Additionally, Group II (CAF+CTG) had a higher mean RC% than Group I (CAF+A-PRF). These results are the following published results about RC% for CAF + PRF [22,24-27]and CAF + CTG [28,29]. However, the reported values of RC % at 6 months for the control group are higher than those reported by da Silva [30] and Jepsen et al. [31] after the treatment of recession defects with CAF + CTG. Those differences in the outcomes could be attributed to the fact that more profound recession defects were treated in the studies by da Silva and Jepsen [30,31].

In the present study, data showed an increase in PPD in the test group at 3 months; however, at 6 months, there was no significant difference in PPD between the two groups (p-value = 0.091). These results suggest that the combination of CAF and A-PRF may have a more significant impact on PPD than CAF and CTG after 3 months, but this effect may not persist over time. This might be attributed to reversible changes in tissue consistency in the test group. In addition, our data showed a significant decrease in CAL for both study groups at 6 months. These data agreed with those published by Jankovic et al. [32]. Based on these data, both A-PRF and CTG are safe and predictable grafting materials in treating localized gingival recessions.

Statistically, WAG values increased significantly in both study groups at 3 and 6 months in the present study, with a higher increase in the control group. However, different results were reported in previous studies in relation to WAG gain after CTG [13,33], an increase in WAG would be expected after CTG. However, very limited studies, if any, reported WAG values after using CAF + A-PRF. The increase in WAG values in both study groups might suggest that both treatment options (CAF + A-PRF and CAF + CTG) could provide comparable stability for at least 6 months.

The mean increase in KTH was statistically significant in both groups at 6 months, with Group II (CAF+CTG) having a higher mean increase than Group I (CAF+A-PRF). These results are consistent with those reported by other investigators [32,34]. This increase in the control group could be explained by the fact that the information in the connective tissue determines the character of the overlying epithelium [34]. In contrast, in the test group, this increase could be explained by the biology of the A-PRF and its growth factors promoting tissue healing and regeneration [19,35].

The results of the present trial also showed that a significantly higher proportion of patients in the control group had a thick biotype at 6 months. This might be explained by the influence of the connective tissue grafting material on the final gingival biotype outcome [34,36].

In addition, the results of the present study suggested that the CAF+CTG group had a more favorable esthetic outcome compared to the CAF+A-PRF group, as reflected by both the RES [37] and VAS-E scores. However, further studies are needed to determine these results' long-term esthetic outcomes and stability.

Moreover, it should be noted that no severe complications, such as bleeding or graft exposure, were reported among the study groups. However, post-operative complications such as pain and swelling have been reported in the control group, and these observations have been confirmed by the findings of other studies [38].

Padma et al. [39] investigated the treatment of localized gingival recessions using CAF alone or CAF combined with PRF and found that the CAF+PRF treatment produced significantly better root coverage compared to CAF alone. On the other hand, Rocuzzo et al. [3] in a systematic review concluded that CAF treatment alone can yield root coverage ranging from 55-91%. However, the present study aimed to evaluate whether the CAF + A-PRF procedure could be an alternative to the CAF+CTG procedure. Therefore, based on the present study's findings, it cannot be concluded how much adding A-PRF improves the results obtained with CAF alone as a standalone treatment.

Combining CAF + A-PRF might provide an appropriate and predictable surgical procedure with minor complications to treat localized recession defects.

The present study has some advantages, including its split-mouth study design, which controls individual patient factors and attains a more precise evaluation of the treatment's impact with a smaller sample size [40]. Overall, these strengths contribute to the validity and reliability of the study results. However, the study also has some limitations, such as the absence of histological evaluations to investigate the potential regenerative capabilities of A-PRF in the treatment of recession defects and the short follow-up period of 6 months. Further research, including histological examinations and longer follow-up periods, is needed to fully understand the effectiveness of A-PRF, including its regenerative capacity on denuded root surfaces and its potential for additional healing over longer periods.

## Conclusions

The present data indicated that the CAF+A-PRF and CAF+CTG techniques could effectively treat localized gingival recessions. However, the combination of CAF and CTG appeared to result in greater improvement in various clinical parameters, such as reduction in recession height and width and mean percentage of root coverage compared to the combination of CAF and A-PRF. Additionally, the use of CTG was associated with a thicker gingival biotype, a higher recession esthetic score, and a higher Visual Analogue Score-Esthetics compared to A-PRF. Using A-PRF in the CAF+PRF technique has the added benefit of avoiding a donor site and reducing patient discomfort after surgery.

In conclusion, CAF+A-PRF may represent an alternative to the traditional CAF+CTG technique; however, further research is needed to confirm these findings and determine the optimal combination of materials for treating gingival recession.
